# Functional Capacity Beyond 350 m in Patients with Chronic Obstructive Pulmonary Disease: A Retrospective Analytical Study

**DOI:** 10.3390/medsci14060663

**Published:** 2026-10-12

**Authors:** Stephanie B. Sant’ana, José J. S. Oliveira, Angela A. Carvalho, Paulo Teixeira, Suelen Nogueira, Olívia Serpa, Raimundo Júnior, Joelma G. Silva, Luís V. F. Oliveira, Mariana P. Sousa, Dante B. Santos

**Affiliations:** 1 Human Movement and Rehabilitation Graduate Program, Evangelical University of Goiás (UniEVANGÉLICA), Anápolis 75083-515, GO, Brazil; 2 Pulmonary and Metabolic Rehabilitation Program, Hospital Universitário de Brasília, Universidade de Brasília, SGAN 604/605, Avenida L2 Norte, Brasília 70840-901, DF, Brazil; 3 Pulmonary Unit, Hospital Universitário de Brasília, Universidade de Brasília, SGAN 604/605, Avenida L2 Norte, Brasília 70840-901, DF, Brazil; 4 Reference Center for Neuromuscular Diseases, Hospital de Apoio de Brasília, AENW 03, Lote A, Setor Noroeste, Brasília 70684-831, DF, Brazil

**Keywords:** chronic obstructive pulmonary disease, six-minute walk test, hypoxemia, spirometry, functional capacity, arterial blood gases

## Abstract

Background: The six-minute walk test (6MWT) in chronic obstructive pulmonary disease (COPD) may be interpreted using either an absolute walking-distance threshold or a percentage of the predicted distance, but these approaches are rarely compared within the same cohort. We examined whether absolute and relative 6MWT criteria classify functional impairment differently and whether they show different associations with resting hypoxemia. Methods: We retrospectively analyzed 75 adults with confirmed COPD who were followed at a tertiary pulmonary outpatient clinic between 2012 and 2018. For the historical categorical classification, the absolute criterion was defined as ≤350 m versus >350 m, and the relative criterion as ≤80% versus >80% of predicted distance after percentage-predicted values had been rounded to the nearest whole percentage. Clinical characteristics, modified Medical Research Council dyspnea score, spirometry, arterial blood gases, 6MWT-derived variables, agreement between criteria, age- and sex-adjusted analysis of covariance, and multivariable logistic regression for hypoxemia (PaO_2_ < 60 mmHg) were evaluated. Results: The absolute criterion identified older and more dyspneic patients, whereas the relative criterion identified patients with greater dyspnea, lower FEV_1_, lower FVC, and lower PaO_2_. Agreement between the criteria was moderate (κ = 0.64; 95% CI 0.48–0.80), and 14 patients who walked >350 m were classified as impaired by the relative criterion. In age- and sex-adjusted logistic regression, both the absolute criterion (OR 2.82; 95% CI 1.02–7.82; *p* = 0.046) and the relative criterion (OR 4.48; 95% CI 1.54–12.98; *p* = 0.006) were associated with hypoxemia. In a sensitivity analysis additionally adjusted for FEV_1_, the relative criterion remained associated with hypoxemia (OR 3.95; 95% CI 1.32–11.79; *p* = 0.014). Conclusions: The absolute and relative 6MWT criteria classified some patients differently and provided partially complementary information. These exploratory findings require prospective validation before either approach is used to guide clinical decision-making.

## 1. Introduction

Chronic obstructive pulmonary disease (COPD) is a major cause of morbidity and mortality worldwide [1] and is increasingly recognized as a multisystem disorder. Beyond persistent airflow limitation, COPD is characterized by small-airway inflammation and remodeling, ventilation–perfusion mismatch, loss of the pulmonary capillary bed, dynamic hyperinflation, systemic inflammation, nutritional impairment, and skeletal muscle dysfunction [2,3,4,5,6]. These abnormalities progressively reduce exercise tolerance and functional capacity [5,6,7,8]. Hypoxemia is particularly relevant because patients with hypoxemic COPD may have worse pulmonary function, lower physical activity, and poorer functional performance [8]. In addition, hypoxemia and hypercapnia activate peripheral and central chemoreflex pathways that modulate ventilatory drive and autonomic responses; altered chemoreflex function may therefore contribute to the complex relationship between gas-exchange abnormalities and exercise limitation in COPD [9]. In low- and middle-income countries, including Brazil, COPD may also arise from heterogeneous exposures beyond cigarette smoking, including household and ambient air pollution and occupational hazards, which may influence disease phenotype and burden [10].

The six-minute walk test (6MWT) is widely used to assess submaximal functional capacity in chronic respiratory disease [11]. In COPD, an absolute walking distance of approximately 350 m has been widely used for prognostic and stratification purposes and has been associated with clinically relevant outcomes, including hospitalization and mortality [12,13,14]. However, a fixed distance does not account for age, sex, height, weight, or population-specific reference equations. Relative interpretation may therefore provide complementary information. Cote et al. evaluated percentage-predicted 6MWT distance in a large multicenter COPD cohort [12], whereas Brazilian data have shown that walking <80% of the predicted distance is associated with a higher risk of acute exacerbations [15]. Brazilian multicenter reference equations are also available for calculating predicted 6MWT distance [16]. Nevertheless, the 80% predicted threshold should not be regarded as a universally validated diagnostic standard for functional impairment; in the present study, it was evaluated as a pragmatic relative criterion.

Despite the clinical use of both approaches, few studies have directly compared absolute and percentage-predicted 6MWT criteria within the same COPD population. This distinction is relevant because the same absolute walking distance may represent different degrees of impairment depending on demographic and anthropometric characteristics, a principle demonstrated in other chronic diseases [17]. Accordingly, this study investigated whether the absolute 350 m criterion and the relative 80% predicted criterion classify the same patients as functionally impaired, quantified disagreement and reclassification between the criteria, and examined their associations with resting hypoxemia and other clinical and pulmonary-function characteristics.

## 2. Materials and Methods

### 2.1. Study Design and Participants

This retrospective analytical study used data from patients with COPD who were followed at a tertiary pulmonary outpatient clinic between February 2012 and February 2018. The study was conducted in accordance with the Declaration of Helsinki and was approved by the Research Ethics Committee of the Faculty of Medicine, University of Brasília (CAAE 94728418.6.0000.5558; Opinion No. 2.872.606; approval date: 4 September 2018). Because the study used retrospective medical-record data, the committee waived the requirement for written informed consent. Verbal consent for participation in the pulmonary rehabilitation program had been documented in the medical records. Data were anonymized before analysis and handled in accordance with the Brazilian General Data Protection Law.

Patients were eligible if they were adults with an obstructive spirometric pattern and a clinical diagnosis of COPD. Exclusion criteria were age > 80 years, refusal to participate, clinical instability, an exacerbation or hospitalization within the previous 3 months, inability to walk without assistance, cognitive impairment that precluded test performance, asthma–COPD overlap, another concomitant lung disease, a history of cancer, or spirometric findings not compatible with COPD.

### 2.2. Clinical, Pulmonary Function, and 6MWT Assessment

Demographic data, body mass index (BMI), comorbidities, and modified Medical Research Council (mMRC) dyspnea scores were extracted from the medical records. Arterial blood gas samples were obtained from the radial artery after 30 min of rest while patients breathed room air. Spirometry was performed by an experienced respiratory technician using a KoKo spirometer (KoKo PFT System, nSpire Health Inc., Longmont, CO, USA). Forced vital capacity (FVC), forced expiratory volume in the first second (FEV_1_), and the FEV_1_/FVC ratio were recorded as absolute values and percentages of predicted values according to established standards and Brazilian reference equations [18,19].

Arterial blood gas sampling and spirometry were performed at least 60 min apart from the 6MWT. All arterial blood gas measurements and 6MWTs included in the analysis were performed while patients breathed room air.

Functional capacity was assessed using the 6MWT in a 30-m unobstructed corridor according to ATS recommendations [11]. Patients were instructed to walk as far as possible for 6 min and were allowed to stop if needed. Walking distance, peripheral oxygen saturation, and heart rate were recorded. Percentage-predicted 6MWT distance was calculated using the Brazilian multicenter reference equation of Britto et al. [16], which incorporates demographic and anthropometric characteristics relevant to the Brazilian population. Derived variables included 6MWT speed (distance/360 s), 6MWT work (body weight × distance), and the distance–saturation product [20,21,22].

All analyzed 6MWTs were performed without supplemental oxygen and without walking aids. A single 6MWT was performed for each patient. No additional familiarization test was conducted because all participants had been previously followed at the service and were already familiar with the 6MWT procedure.

### 2.3. Classification Criteria and Statistical Analysis

Patients were classified according to the operational definitions used in the source dataset: absolute 6MWT distance ≤350 m versus >350 m [13], and relative 6MWT distance ≤80% predicted versus >80% predicted [15,16]. Percentage-predicted distance was calculated using the Brazilian multicenter equation of Britto et al. [16] and rounded to the nearest whole percentage before categorical classification. The 350 m threshold was selected because of its established prognostic and stratification relevance in COPD, whereas the 80% predicted threshold was used as a pragmatic relative criterion based on previous Brazilian COPD data and Brazilian reference equations; it was not considered a universally validated diagnostic standard for functional impairment. The final sample was a convenience sample of all eligible patients with complete data.

Reporting followed the STROBE recommendations [23]. Continuous variables are presented as mean ± standard deviation. Normality was assessed using the Kolmogorov–Smirnov or Shapiro–Wilk test, as appropriate. Between-group comparisons were performed using independent-samples t tests or Mann–Whitney U tests, as appropriate. Agreement between criteria was evaluated using Pearson’s chi-square test, McNemar’s test for paired proportions, Cohen’s kappa with a 95% confidence interval, and observed agreement. Hypoxemia was defined as PaO_2_ < 60 mmHg. Separate multivariable logistic regression models evaluated the associations of the absolute and relative criteria with hypoxemia after adjustment for age and sex. Because FEV_1_ differed between the relative-criterion groups, a sensitivity model additionally adjusted for FEV_1_. With 30 hypoxemic events, the primary three-predictor models provided approximately 10 events per predictor; the four-predictor sensitivity model was therefore considered exploratory. Multicollinearity was assessed using variance inflation factors, which were low in the sensitivity model (all VIFs < 1.13). Model calibration was explored using the Hosmer–Lemeshow test (*p* = 0.12 for the primary relative-criterion model and *p* = 0.34 for the FEV_1_-adjusted sensitivity model). Analysis of covariance (ANCOVA) assessed differences in PaO_2_ by criterion after adjustment for age and sex. No formal correction for multiple comparisons was applied because the analyses were exploratory; exact *p* values are therefore reported and interpreted cautiously. Statistical significance was set at *p* < 0.05. Statistical analyses were performed using the source dataset, and the revised regression estimates were independently verified.

Sensitivity checks identified no relevant multicollinearity. However, influence diagnostics identified several observations with comparatively high leverage, and formal assessments of linearity in the logit suggested possible nonlinearity for some continuous covariates. Because the sample size and number of events did not support more complex spline-based models without increasing the risk of overfitting, the regression estimates should be interpreted as exploratory and in conjunction with their confidence intervals.

## 3. Results

**Figure 1:**
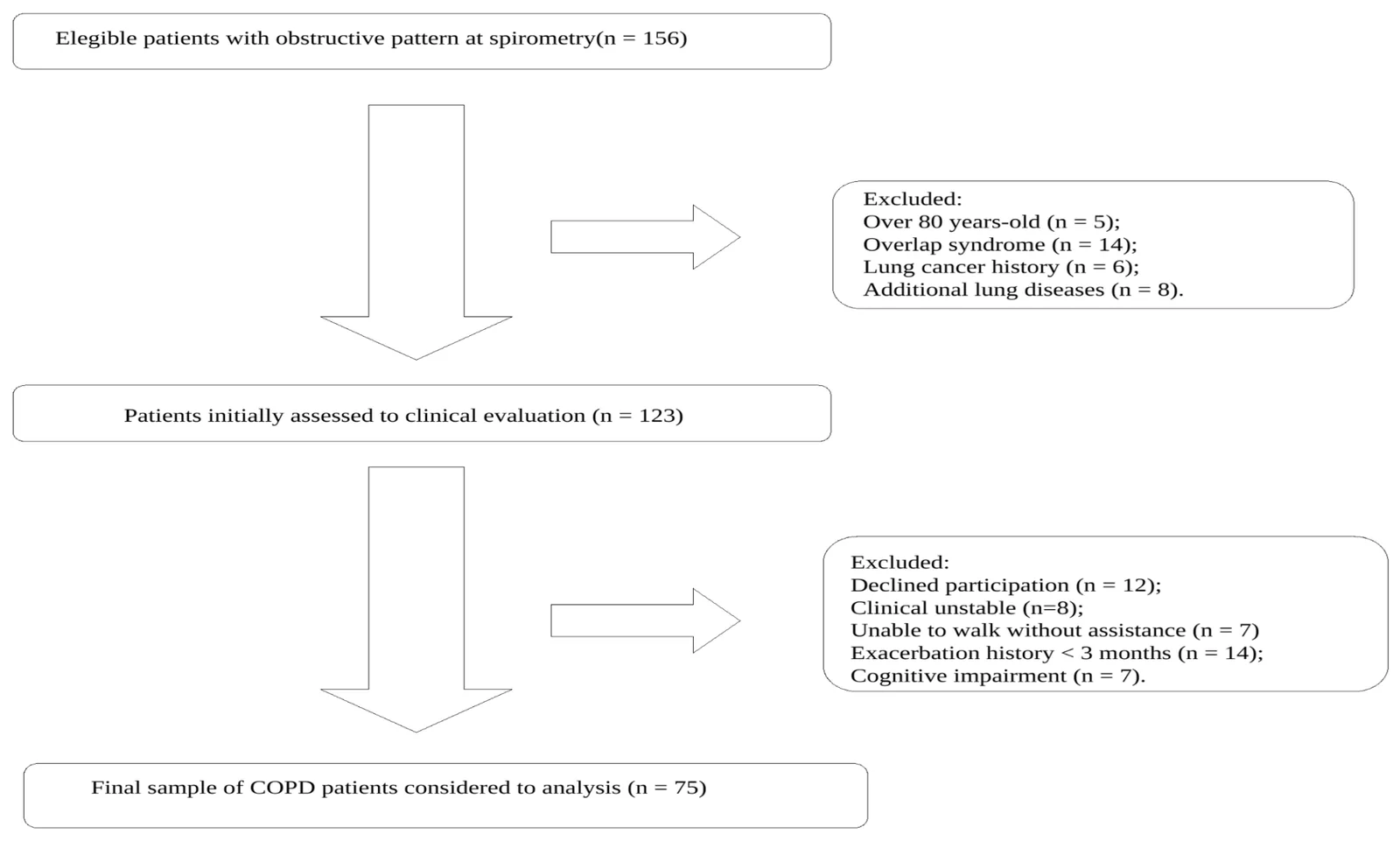
Sample flowchart showing patient selection and the final COPD cohort. Verification of the source worksheets confirmed that one excluded record did not meet spirometric criteria compatible with COPD and two excluded records had been assessed within 30 days after an exacerbation; these records were already encompassed by the exclusion process shown in the flowchart.

Of 156 patients initially screened with an obstructive spirometric pattern, 75 patients with COPD were included in the final analysis (Figure 1). Verification of the source worksheets confirmed that three records were not part of the final analytical cohort: one patient did not meet spirometric criteria compatible with COPD, and two patients had been assessed less than 30 days after a COPD exacerbation. These records were already encompassed by the exclusion process summarized in Figure 1 and were not additional exclusions. The most common comorbidities were systemic hypertension (71/75), heart disease (27/75), diabetes mellitus (27/75), and dyslipidemia (25/75). Two or more comorbidities were present in 36/75 patients classified by the absolute criterion and in 30/75 patients classified by the relative criterion.

Using the absolute criterion, 28 patients walked ≤350 m and 47 walked >350 m. Patients in the ≤350 m group were older and more dyspneic, whereas spirometric variables and arterial blood gases did not differ significantly between the absolute-distance groups. Using the relative criterion, 42 patients were classified as ≤80% predicted and 33 as >80% predicted. Patients in the ≤80% predicted group had higher mMRC scores, lower FEV_1_, lower FVC, and lower PaO_2_ (Table 1).

Thirty patients had resting hypoxemia (PaO_2_ < 60 mmHg). Of these, 16 were in the ≤350 m group and 23 were in the ≤80% predicted group. All 6MWT-derived variables differed significantly between the groups classified as impaired and non-impaired by either criterion (Table 2).

The two criteria were associated but not equivalent. Pearson’s chi-square statistic was 35.1 (*p* < 0.001), Cohen’s kappa was 0.64 (95% CI 0.48–0.80), observed agreement was 81.3%, and McNemar’s test indicated a significant difference in paired classifications (*p* < 0.001). Fourteen patients who walked >350 m were classified as impaired by the relative criterion (Table 3).

Fourteen patients who walked >350 m were classified as impaired by the relative criterion. In this discordant subgroup, mean age was 65.4 ± 6.1 years; 11/14 patients (78.6%) were men; mean BMI was 25.2 ± 4.8 kg/m^2^; mean mMRC score was 3.4 ± 1.2; mean FEV_1_ was 30.7 ± 9.1% predicted; mean FVC was 55.4 ± 11.6% predicted; mean PaO_2_ was 58.2 ± 15.5 mmHg; mean 6MWT distance was 405.6 ± 26.1 m; and mean 6MWT distance was 73.1 ± 3.5% predicted. Seven of these 14 patients (50.0%) had resting hypoxemia (PaO_2_ < 60 mmHg).

Supplementary Table S1 summarizes the characteristics of the 14 discordant patients who walked >350 m but met the relative impairment criterion.

After adjustment for age and sex, ANCOVA showed no significant difference in PaO_2_ according to the absolute criterion (F = 1.25; *p* = 0.27; partial η^2^ = 0.02), whereas PaO_2_ differed significantly according to the relative criterion (F = 5.86; *p* = 0.018; partial η^2^ = 0.08). In multivariable logistic regression adjusted for age and sex, the absolute criterion was associated with hypoxemia (OR 2.82; 95% CI 1.02–7.82; *p* = 0.046), and the relative criterion had a larger odds-ratio estimate (OR 4.48; 95% CI 1.54–12.98; *p* = 0.006). In the sensitivity model additionally adjusted for FEV_1_, the relative criterion remained associated with hypoxemia (OR 3.95; 95% CI 1.32–11.79; *p* = 0.014), whereas FEV_1_ was not independently associated with hypoxemia (OR per 1% predicted increase, 0.98; 95% CI 0.95–1.02; *p* = 0.35). Full model estimates are presented in Table 4.

## 4. Discussion

This study indicates that absolute and relative 6MWT criteria provide related but non-equivalent information in this COPD cohort. The fixed 350 m criterion identified an older and more dyspneic subgroup, consistent with its recognized prognostic value [12,13,14,24,25]. It was not associated with continuous resting PaO_2_ in the age- and sex-adjusted ANCOVA, but it was associated with dichotomous hypoxemia in logistic regression (OR 2.82; 95% CI 1.02–7.82). The relative criterion identified patients with greater dyspnea, lower FEV_1_, lower FVC, and lower PaO_2_ and yielded a larger age- and sex-adjusted odds-ratio estimate for hypoxemia (OR 4.48; 95% CI 1.54–12.98). This association persisted in the exploratory sensitivity model additionally adjusted for FEV_1_ (OR 3.95; 95% CI 1.32–11.79). These findings demonstrate association and differential classification but do not establish clinical superiority of the relative threshold.

A clinically relevant observation is that a patient may walk >350 m yet perform at or below 80% of the predicted distance. In the present cohort, this occurred in 14 patients. These patients had a markedly reduced mean FEV_1_ (30.7% predicted), a mean relative walking performance of 73.1% predicted, and a 50% prevalence of resting hypoxemia. Such discordance is physiologically plausible because percentage-predicted distance incorporates individual characteristics that influence expected exercise performance. However, without an external gold standard for functional impairment, these patients should be described as reclassified by the relative criterion rather than as definitively “missed” by the absolute threshold.

The 6MWT-derived variables were also lower in the groups classified as impaired. Lower gait speed, lower walk work, and lower distance–saturation product have been associated with adverse outcomes in COPD and other respiratory diseases [14,20,21,22,26,27,28,29]. Because these measures are mathematically related to walking distance, their between-group differences should not be interpreted as independent validation of either threshold; rather, they describe the broader functional profile of the classified groups. Percentage-predicted 6MWT performance has also been used to characterize response patterns after pulmonary rehabilitation in COPD [30].

Our findings do not diminish the value of the 350 m threshold. Rather, they suggest that the two approaches capture partially different aspects of functional performance. The relative criterion identified a broader subgroup with worse pulmonary function and lower resting PaO_2_ and yielded a larger odds-ratio estimate for hypoxemia in multivariable analysis, including the FEV_1_-adjusted sensitivity model. Nevertheless, the absolute criterion was also associated with dichotomous hypoxemia in the age- and sex-adjusted logistic model, whereas it was not associated with continuous PaO_2_ in ANCOVA. This divergence, together with the sample size and model diagnostics, argues against claiming clinical superiority of either threshold. Resting hypoxemia is only one contributor to exercise limitation in COPD; ventilatory limitation, dynamic hyperinflation, ventilation–perfusion mismatch, skeletal muscle dysfunction, altered ventilatory drive and chemoreflex responses, cardiovascular comorbidity, and exercise-induced gas-exchange abnormalities may all influence 6MWT performance [5,6,9].

**Figure 2:**
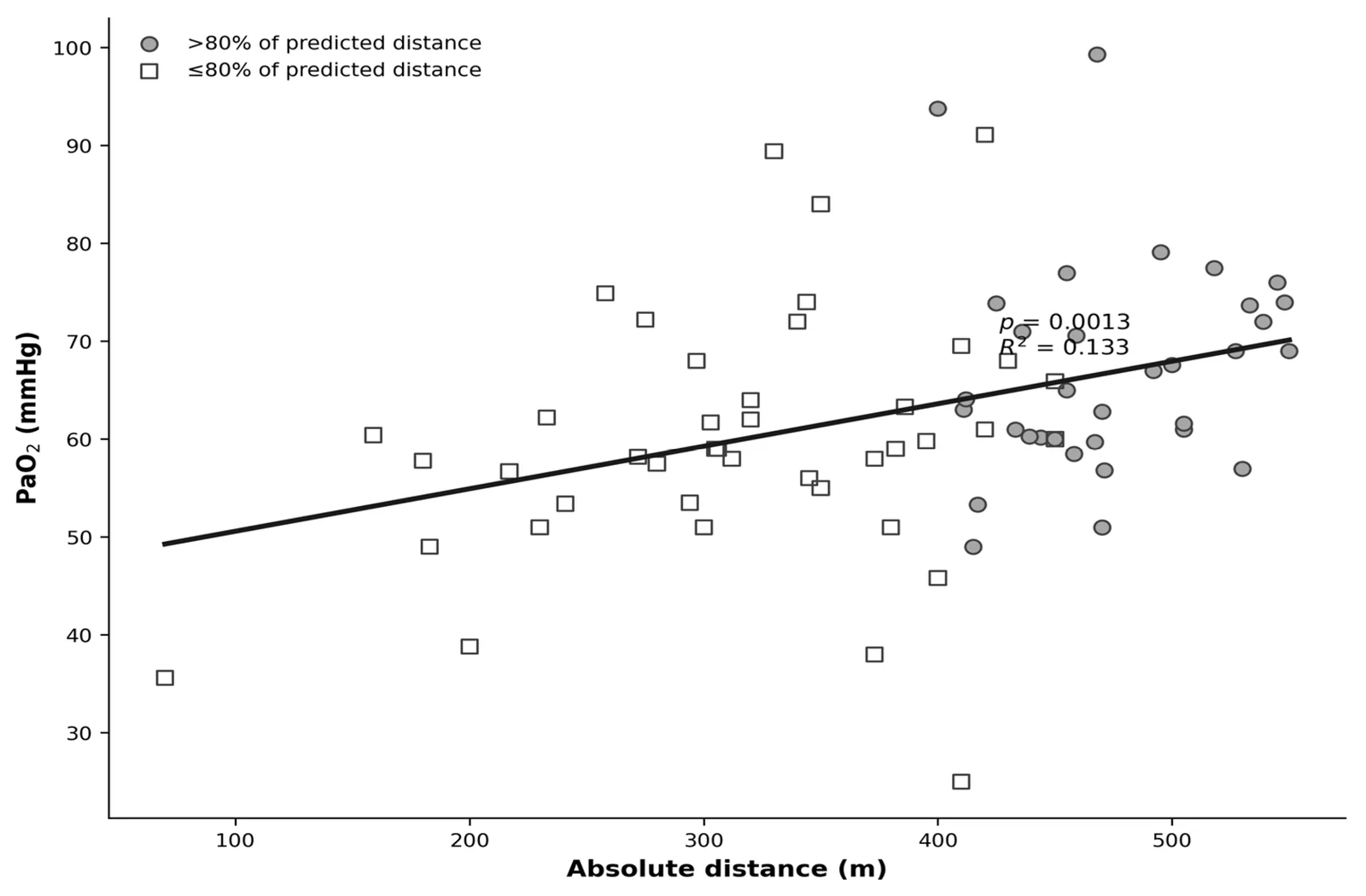
Relationship between arterial oxygen partial pressure and absolute 6MWT distance. Pearson’s r = 0.365 (95% CI 0.150–0.546), *p* = 0.0013; R^2^ = 0.133.

**Figure 3:**
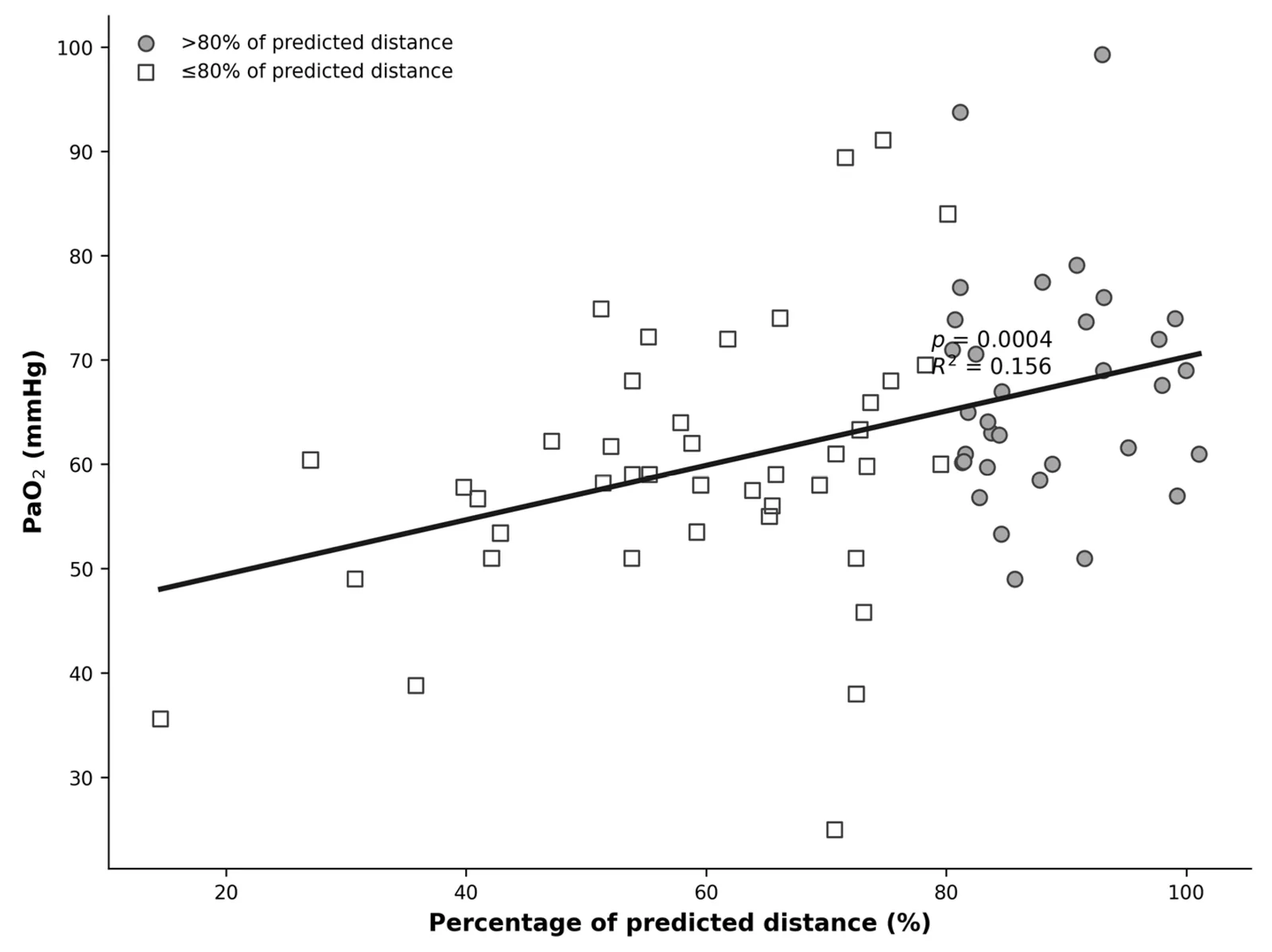
Relationship between arterial oxygen partial pressure and percentage-predicted 6MWT distance. Pearson’s r = 0.396 (95% CI 0.185–0.571), *p* = 0.0004; R^2^ = 0.156.

The high burden of systemic hypertension and cardiovascular disease in this cohort also warrants consideration. COPD-related systemic inflammation and shared cardiometabolic risk factors may compound cardiovascular risk and functional limitation, although the present study was not designed to isolate these pathways. Recent literature has emphasized interactions among COPD phenotypes, prior pulmonary insults, systemic inflammation, and cardiovascular risk [31]. The relationships between resting PaO_2_ and absolute and percentage-predicted 6MWT distances are shown in Figure 2 and Figure 3, respectively.

The correlation between resting PaO_2_ and absolute walking distance was statistically significant but modest; the linear relationship explained 13.3% of the variance.

The correlation between resting PaO_2_ and percentage-predicted walking distance was also statistically significant but modest; the linear relationship explained 15.6% of the variance.

### Limitations

This retrospective, single-center study used a convenience sample, which limits external validity and statistical power. Selection bias is possible because only 75 of 156 initially screened patients were included and complete data were required; therefore, the analyzed cohort may differ systematically from the broader COPD population evaluated at the center. The modest sample size and 30 hypoxemic events constrained multivariable modeling: the primary models included three predictors, whereas the FEV_1_-adjusted four-predictor sensitivity model had only 7.5 hypoxemic events per predictor and should be regarded as exploratory. Influence diagnostics identified several relatively influential observations, and formal assessments of linearity in the logit suggested possible nonlinearity for some continuous covariates; more flexible modeling was avoided because of the limited event count. Resting PaO_2_ was used to define hypoxemia; therefore, isolated exercise-induced desaturation, nadir SpO_2_, and time spent in desaturation during the 6MWT were not captured. All analyzed 6MWTs were performed on room air and without walking aids, and arterial blood gas sampling and spirometry were performed at least 60 min apart from the 6MWT. A single 6MWT was performed per patient; no additional familiarization test was conducted because all participants were already familiar with the procedure through prior follow-up at the service. Medication use and long-term oxygen therapy status were not consistently available in the retrospective records and therefore could not be included as covariates, leaving the possibility of residual confounding. The modest correlations between resting PaO_2_ and walking performance (R^2^ = 0.13–0.16) indicate that arterial oxygenation explains only a limited proportion of variability in functional performance. Prediction equations and spirometric reference values may not generalize to all populations, and the historical categorical classification used rounded percentage-predicted values. No formal correction for multiple comparisons was applied; therefore, nominal *p* values should be interpreted cautiously. Prospective studies are needed to determine whether the observed reclassification improves prognostic assessment or clinical decision-making.

## 5. Conclusions

The absolute and relative 6MWT criteria classified some patients differently in this COPD cohort and may provide complementary descriptive information. The absolute criterion identified older and more dyspneic patients, whereas the relative criterion identified a broader subgroup with worse pulmonary mechanics and lower resting PaO_2_ and reclassified 14 patients who walked >350 m. After adjustment for age and sex, both criteria were associated with dichotomous hypoxemia, with a larger odds-ratio estimate for the relative criterion; the association with the relative criterion persisted in an exploratory sensitivity model additionally adjusted for FEV_1_. These findings do not establish the superiority of either threshold or demonstrate that combined reporting improves clinical decision-making. Prospective validation is required.

## Figures and Tables

**Table 1:** Demographic and pulmonary function evaluation of patients with COPD by absolute and relative 6MWT criteria.

Variable	≤350 m (*n* = 28)	>350 m (*n* = 47)	*p*	≤80% Predicted (*n* = 42)	>80% Predicted (*n* = 33)	*p*
Age	71.1 ± 7.8	67.3 ± 7.2	0.04	69.2 ± 7.7	68.2 ± 7.6	ns
Sex (M/F)	15/13	28/19	ns	26/16	17/16	ns
BMI (kg/m^2^)	27.2 ± 6.0	25.4 ± 4.2	ns	26.5 ± 5.7	25.5 ± 4.0	ns
mMRC score	3.6 ± 0.8	2.7 ± 1.3	<0.001	3.5 ± 0.9	2.4 ± 1.2	0.01
FEV_1_ (%)	41.5 ± 18.0	42.6 ± 18.3	ns	37.9 ± 16.3	47.6 ± 18.9	0.01
FVC (%)	60.4 ± 17.9	64.6 ± 15.0	ns	58.7 ± 16.1	68.5 ± 14.7	0.008
PaO_2_ (mmHg)	60.5 ± 11.7	64.5 ± 12.9	ns	59.8 ± 13.0	67.1 ± 10.9	0.006
PaCO_2_ (mmHg)	36.2 ± 6.8	36.0 ± 4.9	ns	37.0 ± 6.5	34.9 ± 4.0	ns

Abbreviations: BMI, body mass index; COPD, chronic obstructive pulmonary disease; FEV_1_, forced expiratory volume in the first second; FVC, forced vital capacity; mMRC, modified Medical Research Council; ns, non-significant; PaCO_2_, partial pressure of carbon dioxide in arterial blood; PaO_2_, partial pressure of oxygen in arterial blood.

**Table 2:** Six-minute walk test results and derived variables in patients with COPD by absolute and relative 6MWT criteria.

Variable	≤350 m (*n* = 28)	>350 m (*n* = 47)	*p*	≤80% Predicted (*n* = 42)	>80% Predicted (*n* = 33)	*p*
Distance (m)	271.9 ± 67.9	453.6 ± 51.0	<0.001	316.5 ± 85.6	474.0 ± 45.0	<0.001
Distance (% predicted)	53.0 ± 15.0	84.0 ± 9.0	<0.001	60.0 ± 16.0	88.0 ± 6.0	<0.001
6MWS (m/s)	0.76 ± 0.2	1.3 ± 0.1	<0.001	0.88 ± 0.2	1.3 ± 0.1	<0.001
6MWW (m·kg)	19,178.4 ± 7116.1	30,895.3 ± 6701.6	<0.001	22,156.7 ± 7826.2	32,075.6 ± 6875.5	<0.001
DSP (m·%)	235.7 ± 61.4	534.3 ± 64.4	<0.001	318.6 ± 130.1	555.3 ± 63.3	<0.001

Abbreviations: 6MWT, six-minute walk test; 6MWS, six-minute walk speed; 6MWW, six-minute walk work; DSP, distance–saturation product.

**Table 3:** Distribution of patients according to absolute and relative 6MWT criteria.

	≤80% Predicted Distance	>80% Predicted Distance	Total
≤350 m	28	0	28
>350 m	14	33	47
Total	42	33	75

**Table 4:** Multivariable logistic regression models for resting hypoxemia (PaO_2_ < 60 mmHg).

Model/Predictor	B	SE	OR	95% CI	*p*	Interpretation
Absolute criterion model						Age- and sex-adjusted
≤350 m criterion	1.037	0.521	2.82	1.02–7.82	0.046	Associated with hypoxemia
Age, per year	0.050	0.035	1.05	0.98–1.13	0.156	
Male sex	0.441	0.517	1.55	0.56–4.28	0.393	
Relative criterion model						Age- and sex-adjusted
≤80% predicted criterion	1.499	0.543	4.48	1.54–12.98	0.006	Associated with hypoxemia
Age, per year	0.066	0.036	1.07	1.00–1.15	0.066	
Male sex	0.230	0.529	1.26	0.45–3.55	0.664	
Sensitivity model						Relative criterion + age + sex + FEV_1_
≤80% predicted criterion	1.374	0.558	3.95	1.32–11.79	0.014	Association persisted
Age, per year	0.073	0.037	1.08	1.00–1.16	0.047	
Male sex	0.174	0.536	1.19	0.42–3.40	0.745	
FEV_1_, per 1% predicted	−0.015	0.016	0.98	0.95–1.02	0.353	

## Data Availability

The data presented in this study are available upon reasonable request from the corresponding author because the data are not publicly available due to privacy or ethical restrictions.

## Supplementary Materials

The following supporting information can be downloaded at: https://www.mdpi.com/article/10.3390/medsci14060663/s1, Table S1: Characteristics of patients with 6MWT distance >350 m but ≤80% of predicted distance.
