# Supplementary material for: Functional Capacity Beyond 350 m in Patients with Chronic Obstructive Pulmonary Disease: A Retrospective Analytical Study
Source: Med Sci (Basel). 2026 Oct 12;14(6):663. doi: 10.3390/medsci14060663 (PMC13706899; doi:10.3390/medsci14060663)
Supplement: Supplementary file 1 [file medsci-14-00663-s001.zip › medsci-4613415-supplementary.pdf]

Supplementary Table S1. Characteristics of patients with 6MWT distance >350 m and ≤80% of predicted distance.

**Characteristic**	**Discordant subgroup (n = 14)**
Age, years	65.4 ± 6.1
Sex, male/female	11/3
BMI, kg/m^2	25.2 ± 4.8
mMRC score	3.4 ± 1.2
FEV_1, % predicted	30.7 ± 9.1
FVC, % predicted	55.4 ± 11.6
PaO_2, mmHg	58.2 ± 15.5
6MWT distance, m	405.6 ± 26.1
6MWT distance, % predicted	73.1 ± 3.5
Resting hypoxemia, n (%)	7 (50.0%)
